# Suppressive effect of pseudolaric acid B on *Echinococcus multilocularis* involving regulation of TGF-β1 signaling *in vitro* and *in vivo*

**DOI:** 10.3389/fmicb.2022.1008274

**Published:** 2022-11-11

**Authors:** Haijun Gao, Lele Huo, Xiaojin Mo, Bin Jiang, Yanping Luo, Bin Xu, Jingzhong Li, Xingming Ma, Tao Jing, Zheng Feng, Ting Zhang, Wei Hu

**Affiliations:** ^1^National Institute of Parasitic Diseases, Chinese Center for Disease Control and Prevention (Chinese Center for Tropical Diseases Research), NHC Key Laboratory of Parasite and Vector Biology, WHO Collaborating Center for Tropical Diseases, National Center for International Research on Tropical Diseases, Shanghai, China; ^2^School of Basic Medical Sciences, Lanzhou University, Lanzhou, Gansu, China; ^3^Ganzr Tibetan Autonomous Prefecture Center for Disease Control and Prevention, Kangding, Sichuan, China; ^4^National Health Commission Key Laboratory of Echinococcosis Prevention and Control, Tibet Autonomous Region Center for Disease Control and Prevention, Lhasa, Tibet, China; ^5^Department of Microbiology and Microbial Engineering, School of Life Sciences, Fudan University, Shanghai, China

**Keywords:** alveolar echinococcosis, pseudolaric acid B, *Echinococcus multilocularis*, protoscoleces, TGF-β1

## Abstract

*Echinococcus multilocularis*, the causative agent of alveolar echinococcosis (AE), severely threats human health and livestock farming. The first line of chemotherapeutic drug for AE is albendazole, which limits rapid extension of *E. multilocularis* metacestodes, but is rarely curative for AE, with severe side effects in long-term use, thus development of new anti-echinococcal drugs is mandated. Pseudolaric acid B (PAB) has long been used to treat fungal-infected dermatosis, and exerted anti-tumor, -fertility, -angiogenesis, -tubulin and antiparasitic activity. However, the effect of PAB against *Echinococcus* spp. remains unclear. The present study is to understand the effect of PAB against *E. multilocularis in vitro* and *in vivo*, and identify potential anti-echinococcal mechanism, as well as its toxicity. After exposure to PAB at 20 μg/ml, significant reduction of the survival rate and substantial ultrastructural destructions in *E. multilocularis* protoscoleces were observed *in vitro*. Furthermore, the wet weight of *E. multilocularis* cysts in the infected mice was significantly decreased after treatment with PAB (40, 20 or 10 mg/kg) for 12 weeks. Meanwhile, significant increase of both protein and mRNA expression of transforming growth factor beta 1 (TGF-β1) was detected in the serum and liver of the infected mice, whereas PAB administration lowered its expression significantly. The toxicity tests demonstrated that PAB displayed lower cytotoxicity to human liver and kidney cells (HL-7702 and HK-2 cell) with IC_50_ = 25.29 and 42.94 μg/ml than albendazole with IC_50_ = 3.71 and 21.22 μg/ml *in vitro*, and caused lower hepatoxicity and nephrotoxicity in mice than ABZ. Our findings indicated that PAB possesses potent anti-echinococcal effect, with lower toxicity than albendazole, implying a potential chemotherapeutic agent for AE. Additionally, the present study demonstrated that the suppressive effect of PAB on the parasite may involve down-regulation of TGF-β1 signaling.

## Introduction

Alveolar echinococcosis (AE) is a neglected zoonotic parasitosis caused by the larval stage of *Echinococcus multilocularis*. It is a cosmopolitan public health issue posing severe harm to human and livestock health, mainly spreading in Central Asia and Tibetan plateau of Western China, Europe and North America (Deplazes et al., 2017). It was reported that more than 91% of annual new AE cases worldwide occurred in rural communities on the Qinghai-Tibet Plateau of Western China, which causes more than 90% of the global disease burden (Kern et al., 2017). *E. multilocularis* metacestodes often reside in the liver of human and rodents as the intermediate host, and show tumor-like infiltrative growth, leading to liver fibrosis and organ-failure, which is often termed “parasitic cancer” (Wang et al., 2016; Gao et al., 2021). If untreated or insufficiently treated, AE patients prognose a mortality rate of > 90% within 10–15 years after diagnosed (Cheng et al., 2020; Wang et al., 2020). Currently, clinical therapeutic strategies of AE include radical resection, liver transplantation and chemotherapy, in which, drug treatment is indispensable for AE patients before and after the surgical treatment (Stamatakos et al., 2009; Kern et al., 2017). Albendazole (ABZ), a derivative of benzimidazole, is the first choice for treatment of AE, while it disrupts the microtubule polymerization and interferes with its energy metabolism (Horton, 2000; Hemphill et al., 2014). However, even if ABZ limits the rapid extension of *E. multilocularis* metacestodes, the curative effect is poor, and its long-term use produces severe adverse reactions (Hemphill and Muller, 2009). Therefore, development of new and effective treatment drugs for AE is urged.

Pseudolaric acid B (PAB), a diterpene acid extracted from the root of *Pseudolarix kaempferi Gordon*, is a traditional Chinese medicine that has been used for treatment of fungal-infected dermatosis for many years (Zhang et al., 2014; Liu et al., 2017). Increasing evidences indicated that PAB have a wide range of antitumor effects. For example, in a nude mouse experiment, PAB inhibits gastric cancer cell lung metastasis, inducing cell apoptosis by suppressing phosphatidylinositol-4,5-bisphosphate 3-kinases and protein kinase B (PI3K/Akt), ERK1/2 and mitochondrial signaling pathways (Wang D. et al., 2017). In addition, our recent study has demonstrated that PAB blocks hepatocellular carcinoma (HCC) cells proliferation and invasion through inhibiting Notch1/Akt signaling pathway (Gao et al., 2022). Moreover, PAB inhibited immunomodulatory functions in T regulatory cells by reducing the expression of protein kinase B (PKB or Akt) and mitogen activated protein kinases (MAPK; Li et al., 2014; Liu et al., 2017), and a derivant of PAB was also evidenced to boost the expression of TGF-β1 in regulatory T cells (Li et al., 2014). Overall, PAB may be a novel effective candidate agent in treatment of cancer, immune disorders, inflammatory diseases, and immunosuppression, even though the mechanism responsible for PAB exerting the biological functions is still poorly understood. For instance, it was noted that PAB has a strong insecticidal effect described in ancient book-Compendium of Materia Medica (Yao et al., 2014), and later studies found it has a strong anti-parasitic effect on flatworm *Dactylogyrus* in fish (Ji, 2013). More importantly, the excessive disorder of transforming growth factor beta 1 (TGF-β1) and many other signaling pathways, such as Notch/Akt, PI3K/Akt, and MAPKs signaling, were found to cross-drive the initiation and progression of many liver diseases, such as liver fibrosis induced by *Echinococcus* infection (Brehm and Koziol, 2017; Wang Y. et al., 2017; Chen et al., 2022). However, the information of PAB against *E. multilocularis* metacestodes and the underlying anti-echinococcal mechanism remain unclear as yet.

The present study is to explore the anti-echinococcal activity of PAB on *E. multilocularis in vitro* and *in vivo*, and the target signaling regulated by PAB during this process. In addition, the cytotoxicity of PAB in normal liver and kidney cell line *in vitro*, and its sub-acute hepatotoxicity and nephrotoxicity in the mice will be assessed.

## Materials and methods

### Biochemical reagents

PAB, ABZ and ABZ sulfoxide (ABZ-SO, an anthelmintic active form of ABZ) were purchased from Aladdin Industrial Corporation (Shanghai, China) and Sigma-Aldrich (St. Louis, MO, United States); cell culture media were obtained from Gibco (Wisent, Canada); human liver cancer cell line (HepG2 cell) and normal human renal epithelial cell line (HK-2 cell) were purchased from Cell bank of Chinese Academy of Sciences (Shanghai, China), and human normal liver cell line (HL-7702 cell) was warmly presented by Dengfeng Yang from Gangxi Academy of Sciences. Mongolian gerbils were purchased from Zhejiang Academy of Medical Sciences (Hangzhou, China).

### Isolation and culture of *Echinococcus multilocularis* protoscoleces

*E. multilocularis* protoscoleces (PSC) were separated from the metacestodes in *E. multilocularis*-infected Mongolian gerbils, and rinsed with dulbecco’s modified eagle’s medium (DMEM) containing 1% of penicillin–streptomycin (P-S) until the viability of > 97%, and then seeded into 24-well culture plates (120 PSC per well) to be incubated under the condition of 37°C and 5% CO_2_, as described previously (Gao et al., 2021).

### Drug treatment of *Echinococcus multilocularis* protoscoleces *in vitro*

The experimental group was assigned as follows: (i) the vehicle group treated with 0.1% dimethyl sulfoxide (DMSO) (*n* = 3); (ii) the ABZ-SO group treated with ABZ-SO of 40, 20 and 10 μg/ml resolved in 0.1% DMSO (*n* = 3), respectively; (iii) the PAB group treated with PAB of 80, 40, 20, 10, 5, 2, 1 and 0.5 μg/ml resolved in 0.1% DMSO (*n* = 3), respectively. After treatment with different doses of drugs for 48 h, the PSC were stained with 0.4% trypan blue and examined morphologically under an inverted light microscope (BX43, Olympus, Japan). The survival rate of PSC were calculated as follows: PSC survival rate (%) = (live PSC count/total PSC count) × 100, with three independent biological replicates, as described previously (Gao et al., 2021), and half maximal inhibitory concentration (IC_50_) values were calculated from the graph plotted inhibition percentage against the concentration of sample solution using the Graphpad/Prism program version 8.0 (San Diego, CA). Further, the PSC exposed to different compounds were fixed with 4% glutaraldehyde to observe the ultrastructural alterations under scanning electron microscope (SEM, JSM-5600LV, JEOL, Japan) and transmission electron microscope (TEM, HT7800, Hitachi Consumer Marketing, Japan), as described previously (Gao et al., 2021).

### Culture and treatment of *Echinococcus multilocularis* microcyst *in vitro*

After PSC co-cultured with the nurse cell (HepG2 cell) for 4 weeks in complete culture media containing 89% DMEM, 1% P-S and 10% fetal bovine serum (FBS) at 37°C, 5% CO_2_, the microcyst developed from PSC were seeded into 24-well culture plate with 3 microcysts per well. The test groups were assigned as: (i) the vehicle group treated with 0.1% DMSO, (ii) the PAB group treated with PAB of 40 and 20 μg/ml resolved in 0.1% DMSO, respectively. The vitality and morphology of *E. multilocularis* microcysts after treatment with PAB for 1, 2, and 4 weeks were inspected, respectively. On wk. 1, alkaline phosphatase (ALP) activity in the culture supernatant of the microcysts was examined using chemiluminescence method under a multifunctional microplate reader (BioTek, US), according to the manufacturer’s reagent specification (Solarbio Science & Technology Co., Ltd., Beijing, China). On week 4 after treatment with PAB, these microcysts were stained by 0.4% trypan blue to observe the morphological alterations by inverted microscopy.

### *Echinococcus multilocularis* infection and drug treatment *in vivo*

Kunming mice (*n* = 30) were infected with *E. multilocularis* PSC (2000 PSC per mouse) by *in situ* surgical intrahepatic implantation in Specific Pathogen Free (SPF) laboratory for 3 months, and meanwhile, other healthy mice (*n* = 5) were processed with a sham procedure as a negative control group. After 3 months post-infection, the infected mice were divided into: (i) the untreated group (*n* = 5), daily treated with only honey/PBS (1:1 v/v), (ii) the ABZ group (*n* = 5), treated with ABZ daily of 40 mg/kg in honey/PBS (1:1 v/v), (iii) the PAB group (*n* = 5), daily treated with PAB (40, 20, 10 and 5 mg/kg) in honey/PBS (1:1 v/v), respectively, and at the same time, the uninfected mice were only administrated with honey/PBS (1:1 v/v). After oral administration of different drugs for 12 weeks, the wet weight of *E. multilocularis* cysts was weighed, the serum and the liver of the mice were collected to examine the expression of TGF-β1.

### Examination of TGF-β1 protein expression in *Echinococcus multilocularis* PSC by immunofluorescence assay

To examine the expression of TGF-β1 by immunofluorescence (IF) assay, *E. multilocularis* PSC in the vehicle (0.1% DMSO) group and PAB-treated (20 μg/ml) group were rinsed with phosphate buffer saline (PBS), and then fixed with 4% paraformaldehyde, as described previously (Wang et al., 2022). The paraffin-embedded PSC sections were blocked with 20% normal goat serum and incubated with rabbit anti-TGF-β1 antibody (1:100) overnight at 4°C. After reaction with Cy3 conjugated goat anti-rabbit IgG (H + L) (1:200; Servicebio technology Co., LTD., Wuhan, China) and DAPI (1:100; abcam, Cambridge, UK), these slides were imaged under a fluorescence microscope (Olympus, Japan). Mean fluorescence intensity indicating TGF-β1 protein expresssion in the images were calculated using ImageJ software (ImageJ, RRID:SCR_003070), as described previously (Jansen et al., 2016).

### Analysis of TGF-β1 protein expression by ELISA, immunohistochemistry-paraffin, and Western blot assays

TGF-β1 level in the serum of the mice was measured by enzyme-linked immunosorbent assay (ELISA), following the manufacturer’s instructions (CUSABIO, Wuhan, China). The content of TGF-β1 was presented as ng/ml after the subtraction of the appropriate control. Further, to observe the expression of TGF-β1 in *Echinococcus* metacestodes and the mice after treatment with PAB, *E. multilocularis* cysts and the liver tissues were fixed with 4% paraformaldehyde for immunohistochemistry-paraffin (IHC-P) examination, as described previously (Liu et al., 2021). Brifely, the slides coated with *E. multilocularis* cysts and the liver tissues were reacted with rabbit anti-TGF-β1 polyclonal antibody (Bioss Co., Beijing, China) and goat anti-rabbit IgG (ZSGB-BIO, Beijing, China) at the dilution of 1:200 and 1:800, respectively, and then these slides were imaged under a light microscope (Olympus, Japan) (10 fields/group), and the semi-quantitative analysis of TGF-β1 protein expression in each field was performed by ImageJ software (National Institutes of Health, Bethesda, MD, USA), as described previously (Antarianto et al., 2022).

Western blot (WB) was used to examine the TGF-β1 expression, briefly, *E. multilocularis* cysts and the liver tissues of the mice were lysed by tissue homogenizer (Tiangen biotech Co., LTD, Beijing, China), the total protein was extracted using Nuclear and Cytoplasmic Protein Extraction Kit (Yeasen Biotechnology (Shanghai) Co., Ltd.), and the protein concentration was detected using BCA Assay Kit (Servicebio, Wuhan, China), as described previously (Wang et al., 2022). Every sample of 60 μg per lane was separated by 12% SDS-PAGE, and then transferred to a polyvinylidene difluoride membrane, and blocked with 5% non-fat milk for 2 h at room temperature. The membrane was incubated with 1:500 diluted rabbit anti-TGF-β1 antibodies overnight at 4°C, and then with 1:2000 diluted goat anti-rabbit IgG for 2 h at room temperature. In addition, the rabbit anti-β-actin antibody was used as an internal standard at the dilution of 1:300 (Bioss, Beijing, China). Semiquantitative analysis was performed by ImageJ software, as described previously (Jansen et al., 2016).

### Determination of TGF-β1 mRNA expression by real-time quantitative PCR

Total RNA in mice livers and *E. multilocularis* cysts was extracted using TRIzol reagent (Invitrogen, San Diego, USA), and reverse-transcribed into cDNA, referring to the reagent instruction (No. RR036A). The RT-qPCR was performed according to the protocols as described (Liu et al., 2021), and the used primers were as follows: mouse *TGF-*β*1* (forward, 5′-CTTCAATACGTCAG ACATTCGGG-3′; reverse, 5′-GTAACGCCAGGAATTGTTGCTA-3′), mouse *β-actin* (forward, 5′-TTGTTACCAACTGGGACG-3′; reverse, 5′-GGCATAGAGGTCTTTACGG-3′). The semi-quantitative analysis of cDNA was performed by the SYBR Green PCR Master Mix (TaKaRa, Tokyo, Japan), following the manufacturer’s protocols. The relative expression level of *TGF-β1* mRNA was normalized to that of *β-actin*, and the comparison of cycle threshold (CT) values of each group was measured using the 2^–ΔΔCT^ method (Xiao et al., 2022).

### Assessment of cytotoxicity of PAB on human normal hepatocytes and kidney cell *in vitro*

To assess the cytotoxicity of PAB, human normal hepatocyte (HL-7702 cell line) and renal cell line (HK-2 cell) were seeded into 96-well culture plates (5 × 10^3^ cells per well) and 24-well culture plates (2 × 10^4^ cells per well) with complete culture media, and followed by incubation at 37°C and 5% CO_2_ for 6 h. PAB and ABZ-SO at a final concentration of 80, 40, 20, 10, 5, 2, 1, 0.5, 0.2 and 0 μg/ml were added into the cell culture plates, respectively (*n* = 6). After treatment with PAB and ABZ-SO for 48 h, cell counting kit-8 (CCK-8) solution (10 μl per well) was added into 96-well culture plates to continuously incubate for 2 h, and the optical density was read under a multifunctional enzyme marking instrument (BioTek, US), and then cell viability was calculated using the following formula: cell viability = [(OD_treatment_ − OD_blank_)/(OD_control_ − OD_blank_)] × 100%, and IC50 values were calculated using the Graphpad/Prism software. In addition, cell morphologic alterations in 24-well culture plates were observed after staining with 1% crystal violet (Solarbio Co., Ltd., Beijing, China) under the indicated inverted microscope. These experimental processes were conducted with three independent biological replicates following the manufacturer’s specifications (Beyotime Biotechnology Co., Ltd., Shanghai, China).

### Evaluation of sub-acute hepatotoxicity and nephrotoxicity of PAB in mice

To evaluate the sub-acute hepatotoxicity and nephrotoxicity of PAB *in vivo*, 15 healthy Kunming mice were assigned as: (i) the control group (*n* = 5), orally administrated with honey/PBS daily (1:1 v/v), (ii) the PAB group (*n* = 5), orally administrated with PAB at 40 mg/kg in honey/PBS daily (1:1 v/v), and (iii) the ABZ group (*n* = 5), orally administrated with ABZ at 100 mg/kg (the dosage of 136.3 mg/kg for mice calculated by the recommended dosage for human with 10 mg/kg; Kern et al., 2017) in honey/PBS daily (1:1 v/v). After treatment with PAB and ABZ for 6 weeks, those mice sera were collected to detect 11 biochemical indexes that were related to the liver and kidney function by chemiluminescent immunoassay (CLIA). At the same time, the liver and kidney tissues of those mice were fixed with 4% paraformaldehyde to observe the pathological alterations by hematoxylin–eosin (HE) staining.

### Statistics

The experimental data were described as the mean ± standard deviation (mean ± SD). The different reading in fluorescence intensity between two groups was analyzed by student’s *t*-test, and the differences in cysts weight, TGF-β1 level, and biochemical indexes among three or more groups were assessed by one-way ANOVA with multiple comparisons. Statistical analysis was performed using GraphPad Prism version 8.0 (San Diego, CA) and SPSS version (IBM, Chicago, IL). *p* < 0.05 indicates significant difference.

## Results

### Effect of PAB on the activity of *Echinococcus multilocularis* protoscoleces *in vitro*

**Figure 1:**
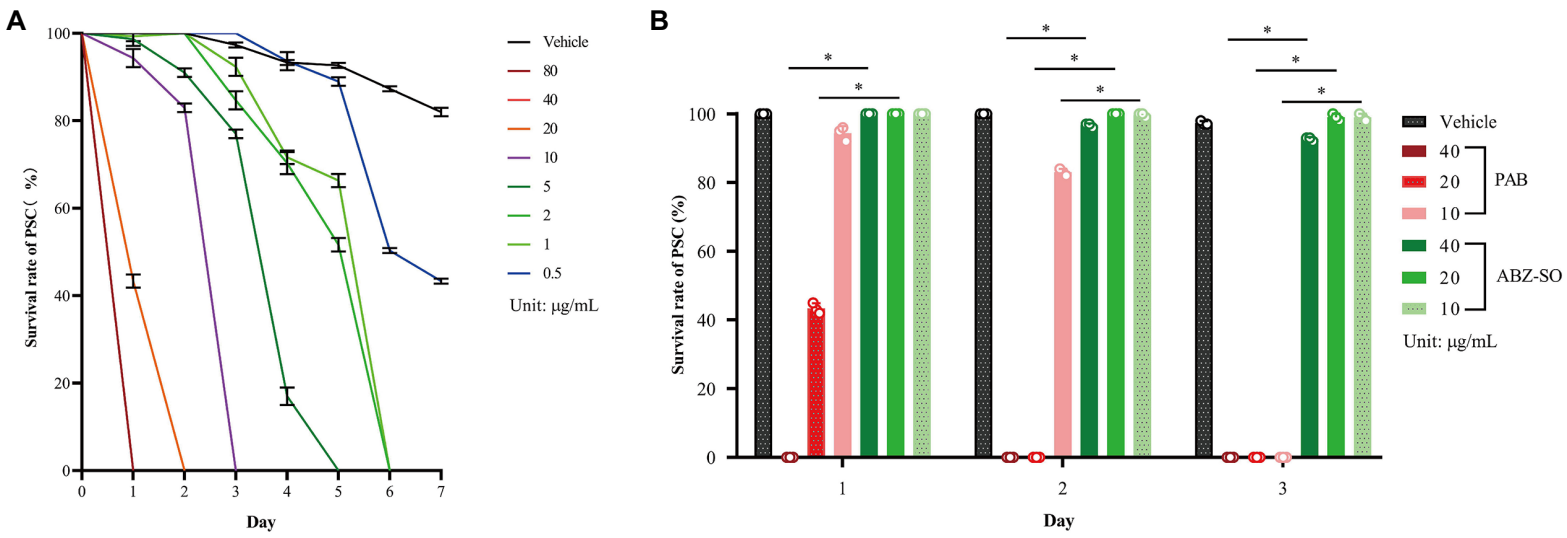
Changes in survival rate of *E. multilocularis* PSC before and after PAB treatment *in vitro*. **(A)** Survival rate of *E. multilocularis* PSC of the vehicle group exposed to 0.5–80 μg/ml PAB for 7 days, and **(B)** 10, 20 and 40 μg/ml PAB and ABZ-SO for 3 days, as measured by morphological alterations under a light microscope. Data are expressed as mean ± SD, *n* = 3. The independent experiments were processed in triplicate.

To investigate insecticidal effect of PAB on *E. multilocularis in vitro*, the survival rate and morphological alterations in PSC were measured after treatment with PAB at different concentrations. The survival rate of PSC showed a time- and dose-dependent decreasing after treatment with 0.5–80 μg/ml PAB within 7 days. All PSC examined were killed by 1–80 μg/ml PAB in 1 week. Among them, all PSC were killed after treatment with PAB at 10, 20 and 40 μg/ml for 3, 2 and 1 day, respectively (Figure 1A). Treatment with PAB (IC_50_ = 12.63 μg/ml) at 40, 20 and 10 μg/ml for 3 days significantly reduced the PSC survival rate in comparison with ABZ-SO treatment at the same concentrations, respectively (all *p* < 0.0001) (Figure 1B).

**Figure 2:**
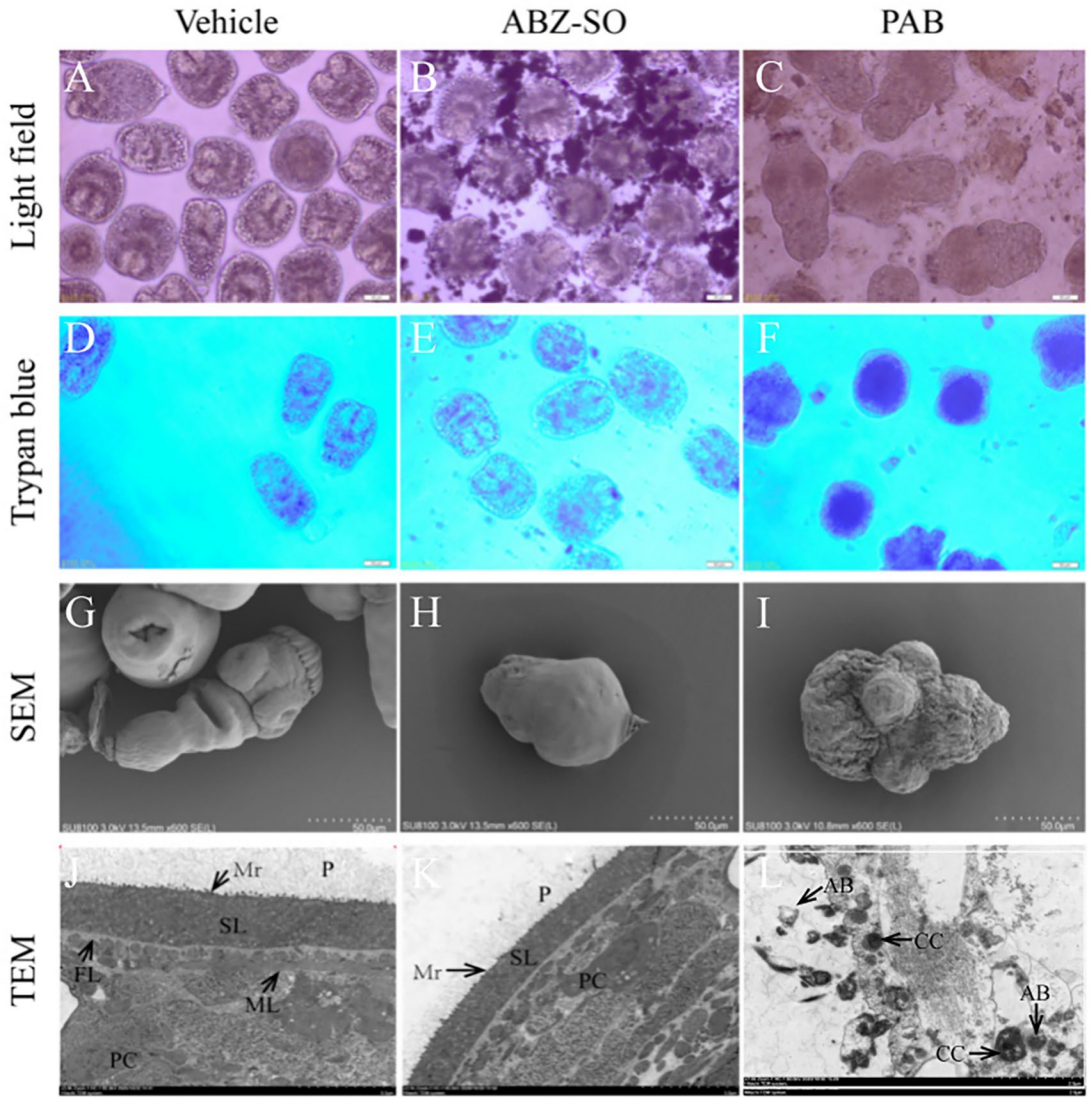
Structural morphology changes of *E. multilocularis* PSC after treatment with PAB *in vitro*. The PSC treated with PAB (20 μg/ml) and ABZ-SO (20 μg/ml) for 2 days were observed using trypan blue staining **(D–L)** or unstained **(A–C)** under light microscope **(A–F)**, 200 × magnification, SEM **(G–I)**, scale-bars: 50 μm and TEM **(J–L)**, scale-bars: 5 μm. Abbreviations: P, periodic acid-schiff stain positive material; Mv, microvillus; SL, syncytial layer, FL, fibrous layer, ML, muscular layer, AB, apoptotic bodies, CC, condensed chromatin.

Light microscopy indicated that structural damage was observed in the PSC after treatment with 20 μg/ml PAB, showing disappearance of calcareous corpuscles, breakage of tegument and suckers, but not seen in the vehicle group with typan blue staining (Figures 2D–F) or not (Figures 2A–C). Furthermore, SEM assay revealed that the ultrastructure of PSC treated with 20 μg/ml PAB was different from that treated with 20 μg/ml ABZ-SO or DMSO. The PSC exposed to 20 μg/ml PAB exhibited ultrastructural destructions, including shedding of tegument, breakage of rostellum and suckers (Figures 2G–I). At the same time, TEM assay indicated that in comparison with the microstructure of PSC treated with ABZ-SO and DMSO, the PSC exposed to PAB showed disappearance of periodic acid-Schiff stain (PAS) positive materials, microvillus and syncytial layer (SL), contraction of parenchymal cells, and presentation of abundant cytoplasm vacuolization, condensed chromatin and apoptotic bodies (Figures 2J–L).

### Effect of PAB on the development of *Echinococcus multilocularis* protoscoleces *in vitro*

**Figure 3:**
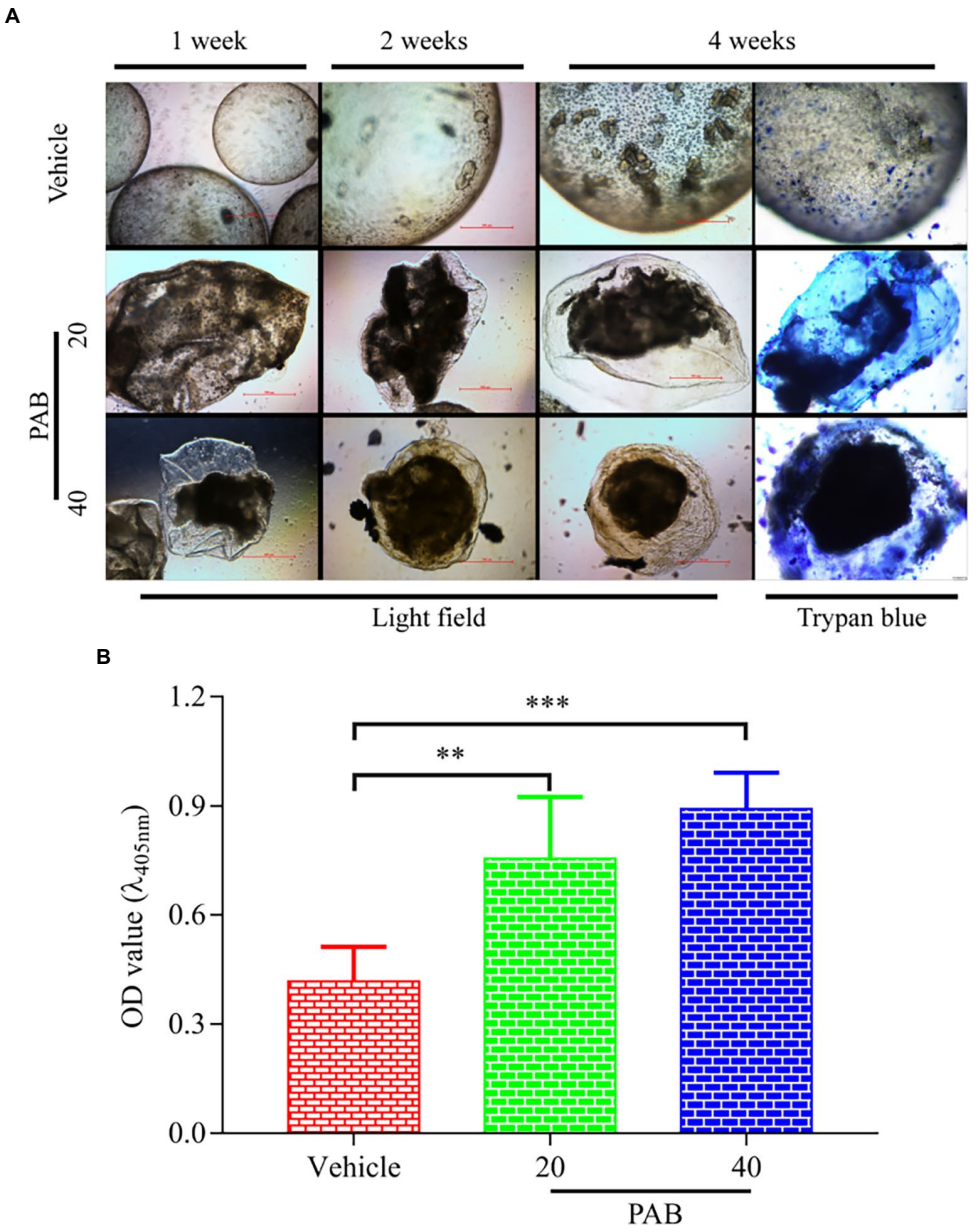
Viability changes of *E. multilocularis* microcysts after treated with PAB *in vitro*. **(A)** Microcysts exposed to 20 and 40 μg/ml PAB for 4 weeks showed ultrastructural damage, such as detachment of the germinal layer from the laminated layer, however the typical germinal layer (GL) (week 1) producing solid protrusions (SP) (week 2), present of daughter PSC (week 4) in the vehicle group. Images were measured by light microscopy at weeks 1, 2 and 4 without any staining; and at week 4 with trypan blue staining. **(B)** ALP levels in the culture supernatants of *E. multilocularis* microcysts after treatment with 20 and 40 μg/ml PAB for 1 week, as measured by chemiluminescence method. All data were assessed using One-way ANOVA with multiple comparisons. The independent experiments were processed in triplicate.

To further investigate anti-echinococcal effect of PAB *in vitro*, the development of *E. multilocularis* PSC after treatment with PAB was measured. Microscopic observation at week 1 of *in vitro* test found that, *E. multilocularis* microcysts upon exposure to PAB of 20 and 40 μg/ml showed morphological alterations, including collapse of the microcyst, shrinking of the germinal layer from the laminated layer, but not in the vehicle group. At weeks 2 and 4, the solid protrusions (SP) and new PSC in the microcysts were observed in the vehicle group, but the microcysts treated with PAB exhibited breakage. At week 4, *E. multilocularis* microcysts after treatment with PAB were found stained by trypan blue and no new PSC was found inside (Figure 3A). In addition, after treatment with 40 and 20 μg/ml PAB for 1 week, the activity of ALP in the culture supernatant of the microcysts exhibited significant increase from (0.42 ± 0.04) U/L in the vehicle group to (0.76 ± 0.08) U/L and (0.89 ± 0.04) in 20 and 40 μg/ml PAB-treated groups, respectively (*p* = 0.0002 and *p* = 0.0085; Figure 3B).

### Effect of PAB against *Echinococcus multilocularis* metacestodes *in vivo*

Furthermore, *in vivo* anti-echinococcal effect of PAB was evaluated in a mouse model with liver echinococcosis. The wet weight of *E. multilocularis* cysts isolated from the group treated with 40, 20 and 10 mg/kg PAB was (1.94 ± 0.30) g, (2.38 ± 0.22) g and (2.64 ± 0.48) g, respectively, which were significantly lower than those from the infected group [(3.82 ± 0.44) g] (*p* < 0.0001, *p* < 0.0001 and *p* = 0.0002), respectively, but not seen in 5 mg/kg PAB treatment group [(3.16 ± 0.37) g] (*p* = 0.0597). In addition, the wet weight of the cysts from 40 mg/kg ABZ treatment group [(2.29 ± 0.15) g] was significantly lower than that in the infected group [(3.82 ± 0.44) g] (*p* = 0.011), but no significant difference was seen when compared with the 40 mg/kg PAB treatment group (*p* = 0.6091; Table 1).

**Table 1:** Changes in the wet weight of *E. multilocularis* metacestodes in mice orally treated with PAB for 12 weeks after 3 months post-infection.

Group	No. of mice	Dose	Cyst weight (g) (mean ± SD)
Untreated	5	5% honey/PBS	3.82 ± 0.44
ABZ	5	40 mg/kg	2.29 ± 0.15^*^
PAB	5	40 mg/kg	1.94 ± 0.30^*^
	5	20 mg/kg	2.38 ± 0.22^*^
	5	10 mg/kg	2.64 ± 0.48^*^
	5	5 mg/kg	3.16 ± 0.37

ABZ, albendazole; PAB, pseudolaric acid B; PBS, phosphate buffer saline. All data were analyzed using one-way ANOVA with multiple comparisons.

^*^p < 0.05 (compared to the untreated group).

### PAB down-regualting TGF-β1 protein and mRNA expression in *Echinococcus multilocularis* metacestodes

**Figure 4:**
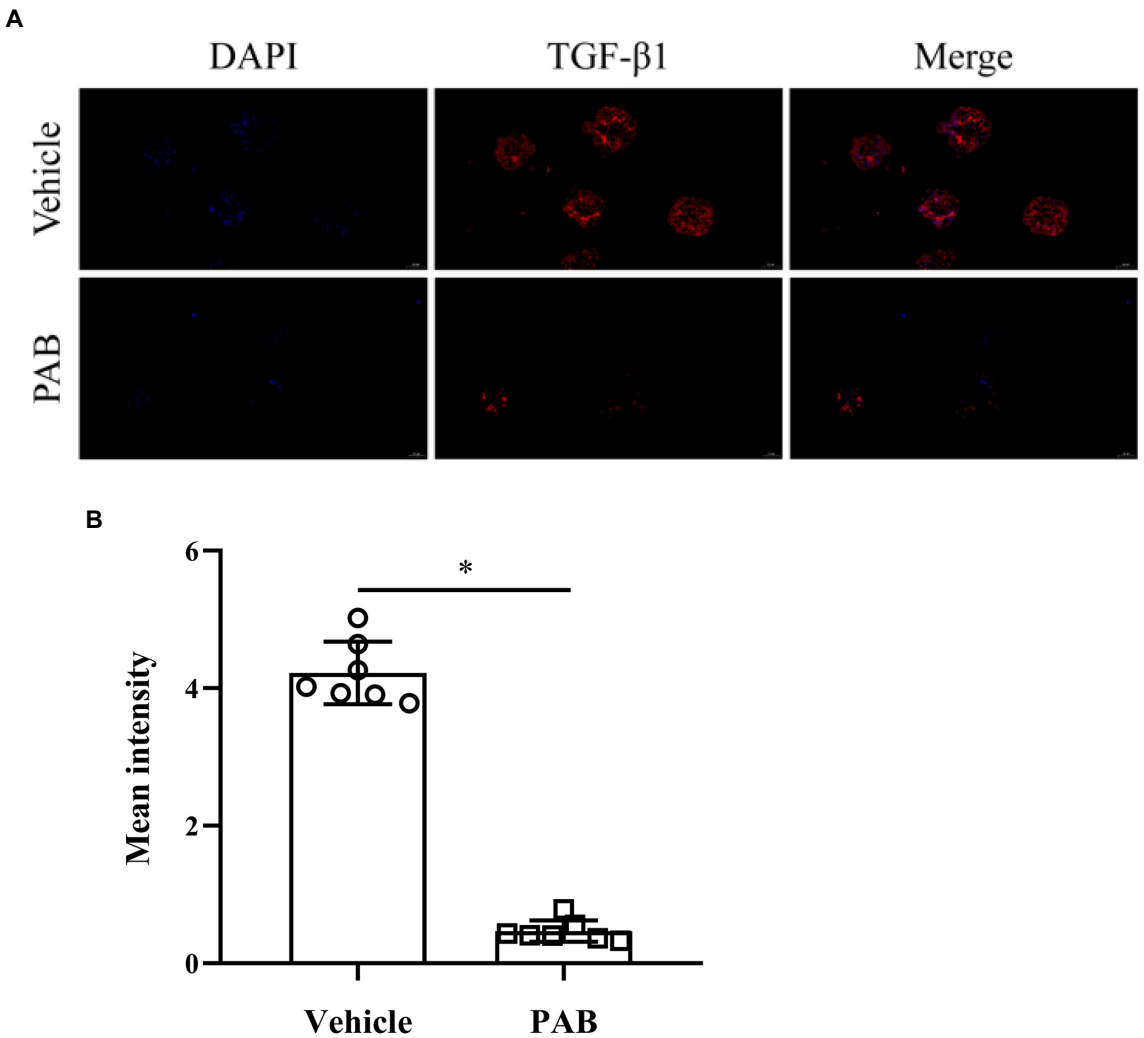
Expression of TGF-β1 in *E. multilocularis* PSC after treatment with 20 μg/ml PAB for 2 days by immunofluorescence assay. **(A)** Location of TGF-β1 protein (red) and DAPT-indicated DNA (blue) in the PSC in immunofluorescence staining images. Scale-bars: 50 μm. **(B)** Analysis of mean fluorescence intensity indicated TGF-β1 protein expression, as measured by ImageJ software. The data was assessed using *t*-test.

Possible anti-echinococcal mechanism of PAB was investigated by observing the expression of TGF-β1 protein and mRNA in *E. multilocularis.* By immunofluorescence assay, red fluorescence-stained TGF-β1 was found widely distributed in the natural parenchymatous tissue of the PSC, but the distribution was diminished after treatment with 20 μg/ml PAB *in vitro* (Figure 4A), and the semiquantitative analysis indicated that compared with the vehicle group, PSC in the PAB-treated group showed a significant decrease of fluorescence-labeled TGF-β1 (*p* < 0.0001; Figure 4B).

**Figure 5:**
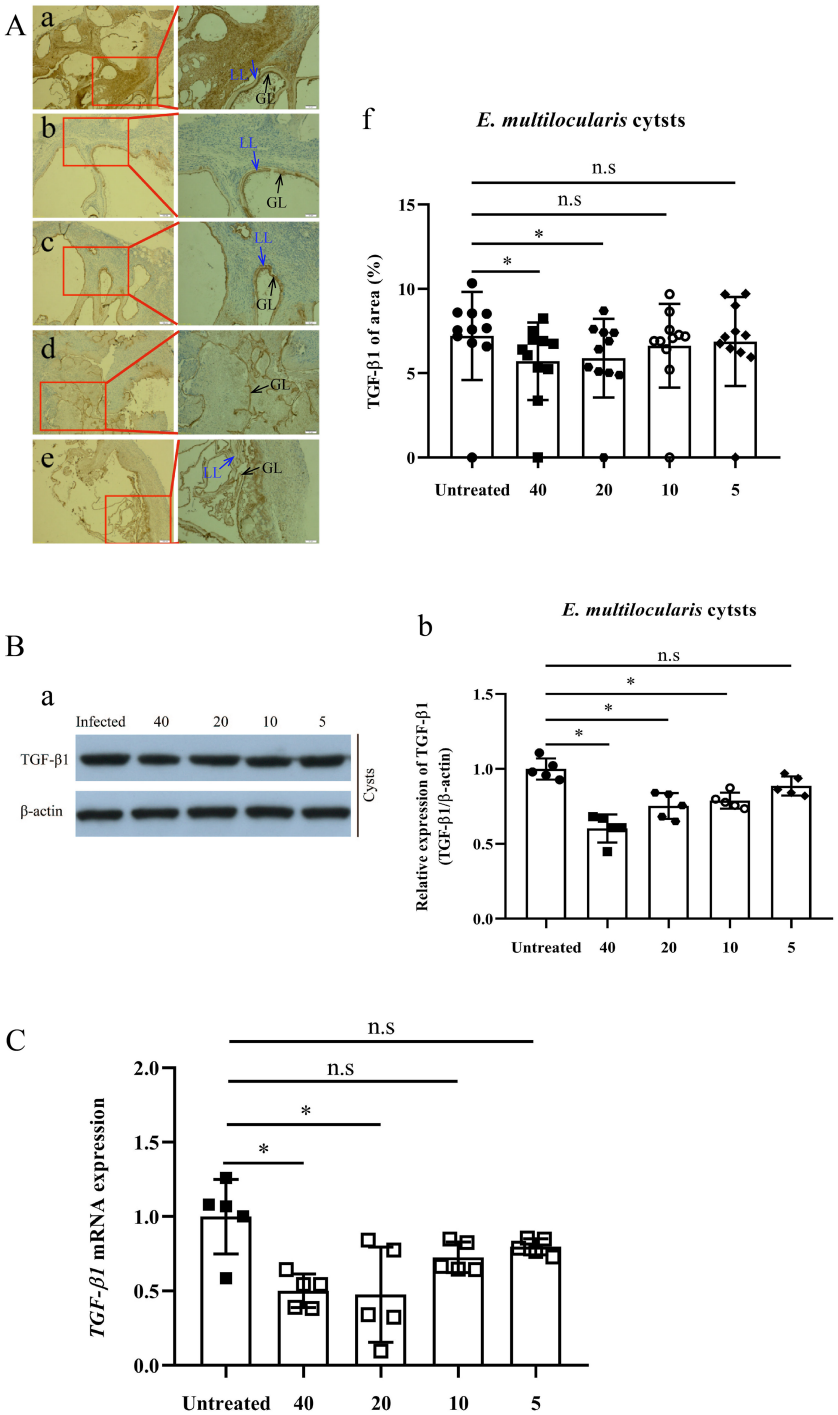
Expression of TGF-β1 protein and mRNA in *Echinococcus multilocularis* cysts after treatment with PAB for 12 weeks, as measured by immunohistochemistry-paraffin (IHC-P), Western blot (WB) and real-time quantitative PCR (RT-qPCR) assay. **(A)** Images of TGF-β1 protein expression in *E. multilocularis* cysts from the mice **(a)** untreated, and treated with PAB **(b)** 40, **(c)** 20, **(d)** 10, and **(e)** 5 mg/kg, as measured by IHC-P assay; and **(f)** semi-quantitative analysis of TGF-β1 protein, as measured by ImageJ software. Brown area indicates expression of TGF-β1 protein, Laminated layer (LL, blue arrow) and germinal layer (GL, black arrow) in *E. multilocularis* cysts. Magnification, × 100 and × 200. **(B)** WB analysis of **(a)** TGF-β1 and β-actin expression in *E. multilocularis* cysts in the untreated, and treated with PAB of 40, 20, 10, and 5 mg/kg PAB mice, and **(b)** the semi-quantitative analysis of TGF-β1 protein, as measured by ImageJ software. **(C)** Analysis of TGF-β1 mRNA expression in *E. multilocularis* cysts by RT-qPCR. All data were assessed using One-way ANOVA with multiple comparisons. LL, laminated layer; GL, germinal layer.

IHC-P assay revealed that the expression of TGF-β1 in *E. multilocularis* cysts [(6.29 ± 1.37)% and (6.29 ± 1.37)%] exhibited a significant decrease after treatment with 40 and 20 mg/kg PAB (*p* = 0.0211 and *p* = 0.0499), respectively, but 10 and 5 mg/kg of PAB caused no change [(7.30 ± 1.21)% and (7.58 ± 1.40)%] (*p* = 0.6247 and *p* = 0.9168) (Figures 5Aa–f), in comparison with that in the infected mice [(7.95 ± 1.10)%].

Furthermore, WB assay showed that the expression of TGF-β1 in *E. multilocularis* cysts [(0.60 ± 0.04), (0.75 ± 0.04) and (0.79 ± 0.02)], were significantly lower after treatment with 40, 20 and 10 mg/kg of PAB (all *p* < 0.0001), respectively, than those in the PAB-untreated mice (1.00 ± 0.03), but no significant reduction was observed at 5 mg/kg of PAB (0.87 ± 0.06) (*p* = 0.0649; Figures 5Ba–b).

Detected by RT-qPCR, expression of *TGF-β1* mRNA in the cysts of infected mice with 40 and 20 mg/kg PAB were reduced to 54.49% and 32.40% (*p* = 0.0024 and *p* = 0.0015), respectively, comparing with the non-treated infected group, but the expression in the group treated with 10 and 5 mg/kg of PAB only showed mild reduction at 64.80% and 84.91% (*p* = 0.1196 and *p* = 0.3189; Figure 5C).

### PAB down-regualting TGF-β1 protein and mRNA expression in the liver of *Echinococcus multilocularis*-infected mice

Furthermore, the expression of TGF-β1 protein and mRNA in *Echinococcus-*infected mouse was also assessed after treatment with PAB. Detected by ELISA, the level of TGF-β1 protein in the serum of the mice was elevated 1.7-fold after infection of *E. multilocularis* (*p* < 0.0001). However, compared with that of the uninfected group, TGF-β1 level in the serum of the infected mice after treatment with 40, 20 and 10 mg/kg of PAB were reduced by 1.9-fold, 1.6-fold and 1.3-fold (*p* < 0.0001, *p* < 0.0001 and *p* = 0.0035), respectively, but 5 mg/kg of PAB treatment group only showed 1.1-fold decrease (*p* = 0.2239; Table 2).

**Table 2:** Changes of TGF-β1 content in the serum of *E. multilocularis-*infected mouse orally treated with PAB for 12 weeks after 3 months post-infection.

Group	No. of mice	Dose	TGF-β1 content (ng/ml, Mean ± SD)
Control	5	5% honey/PBS	13.78 ± 2.16
Untreated^a^	5	5% honey/PBS	23.73 ± 2.56^*^
PAB^b^	5	40 mg/kg	12.08 ± 2.22^*^
	5	20 mg/kg	14.67 ± 1.98^*^
	5	10 mg/kg	18.08 ± 3.10^*^
	5	5 mg/kg	20.89 ± 1.67

PAB, pseudolaric acid B; TGF-β1, transforming growth factor beta 1. ^a^Compared with the control group, and ^b^compared with the untreated group. All data were analyzed using one-way ANOVA with multiple comparisons.

* *p* < 0.05.

**Figure 6:**
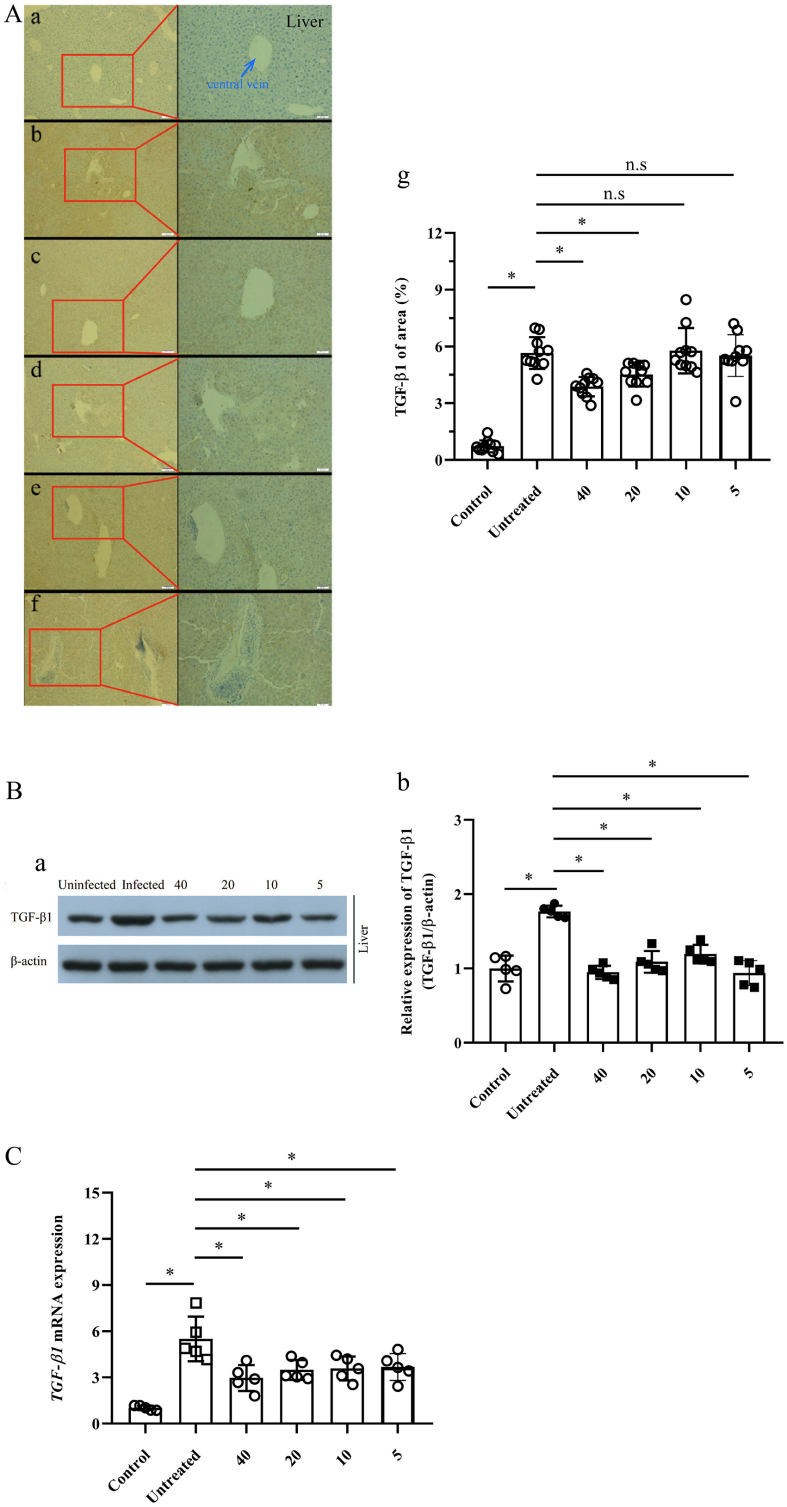
Expression of TGF-β1 protein and mRNA in the liver of *Echinococcus*-infected mouse after treatment with PAB for 12 weeks, as measured by IHC-P, WB and RT-qPCR assay. **(A)** Images of TGF-β1 protein expression in the liver of the mice, **(a)** uninfected, **(b)** untreated, and treated with PAB of **(c)** 40, **(d)** 20, **(e)** 10 and **(f)** 5 mg/kg, as measured by IHC-P assay; and **(g)** the semi-quantitative analysis of TGF-β1 protein, as measured by ImageJ software. Brown area indicates expression of TGF-β1 protein, central vein (blue arrow) in the liver of *E. multilocularis*-infected mice. Magnification, × 100 and × 200. **(B)** WB analysis of **(a)** TGF-β1 and β-actin expression in the mice liver, and **(b)** the semi-quantitative analysis of TGF-β1 protein, as measured by ImageJ software. **(C)** Analysis of TGF-β1 mRNA expression in the liver of the mice by RT-qPCR. All data were assessed using One-way ANOVA with multiple comparisons.

Furthermore, IHC-P assay indicated that the expression of TGF-β1 in the mice liver was boosted fivefold after the infection of *E. multilocularis* (*p* < 0.0001). However, the expression of TGF-β1 in the infected mice treated with 40 and 20 mg/kg PAB was decreased significantly (*p* < 0.0001 and *p* = 0.0167), whilst no significant reduction was found in 10 and 5 mg/kg PAB groups (*p* = 0.999 and *p* = 0.9979; Figures 6Aa–g).

Western blot revealed that the TGF-β1 expression in the liver of *E. multilocularis* infected mouse was significantly increased (*p* < 0.0001), but significantly decreased after treatment with PAB of 40, 20, 10 and 5 mg/kg (all *p* < 0.0001), respectively (Figures 6Ba–b).

In addition, RT–qPCR indicated that the expression of *TGF-β1* mRNA in the liver of the mice was elevated 5.5-fold after infection of *E. multilocularis* (*p* < 0.0001). However, compared with that in *Echinococcus*-infected mice, RT-qPCR indicated that the expression of *TGF-β1* mRNA in the liver of the infected mice treated with 40, 20, 10 and 5 mg/kg of PAB was decreased to be 2.9-, 3.5-, 3.6- and 3.7-folds (*p* = 0.0006, *p* = 0.0059, *p* = 0.012 and *p* = 0.0136), respectively (Figure 6C).

### Hepatorenal cytotoxicity of PAB *in vitro*

**Figure 7:**
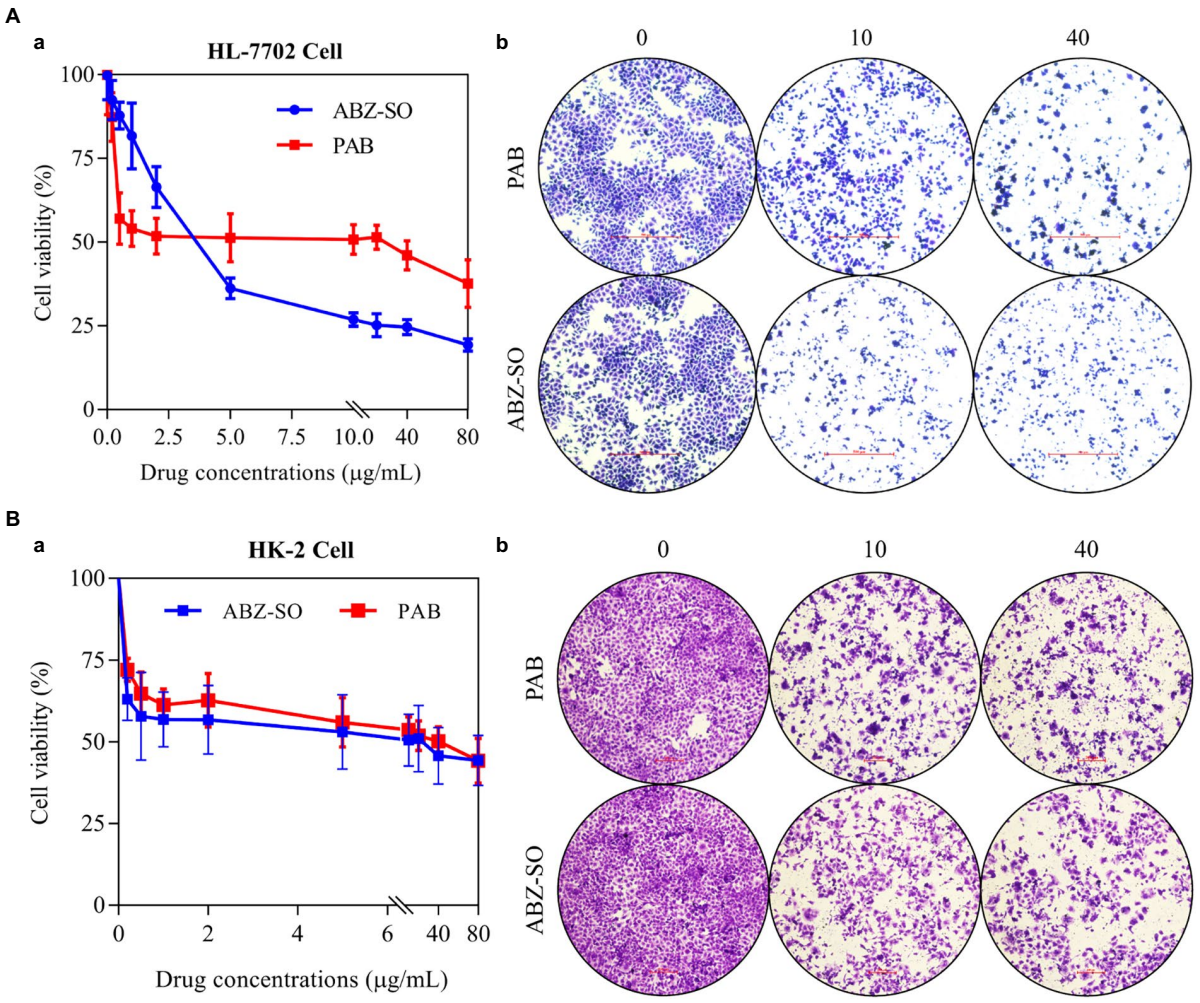
Cytotoxicity of PAB on human hepatocytes and renal cells *in vitro*. The cell livability **(a)** and number **(b)** of HL-7702 cell **(A)** and HK-2 cell **(B)** after treatment with different concentrations of PAB and ABZ-SO (0–80 μg/ml) for 48 h by CCK-8 assay **(A)** and crystal violet staining **(B)**. The independent experiments were processed in triplicate.

To assess the toxicity of PAB, *in vitro* the proliferation and morphology of the normal liver and kidney cell were first measured using cell counting kit (CCK-8) assay and crystal violet staining. CCK-8 indicated the survival rates of HL-7702 cell showed a dose-dependent decrease after treatment with different concentrations of PAB and ABZ-SO at 0.2, 0.5, 1, 2, 5, 10, 20, 40 and 80 μg/ml (Figure 7Aa). Among them, treated with 10 μg/ml PAB (IC_50_ = 25.29 μg/ml), the cell survival rate showed a significant increase [(50.79 ± 1.80)%] in comparison with that [(26.9 ± 0.81)%] using the same concentration of ABZ-SO (IC_50_ = 3.71 μg/ml) (*p* = 0.0001). When the concentrations of PAB and ABZ-SO reached 40 μg/ml, the cell survival rate in PAB-treated group [(46.07 ± 1.77)%] was also significantly higher than that in ABZ-SO-treated group [(24.65 ± 0.93)%] (*p* = 0.0001). In addition, crystal violet staining assay showed reduction in the number of viable HL-7702 cells after treated with 10 and 40 μg/ml PAB or ABZ-SO. Among them, ABZ-SO presented a stronger inhibitory effect than the same concentrations of PAB (Figure 7Ab).

Furthermore, CCK-8 assay indicated dose-dependent inhibitory effect of both PAB and ABZ-SO at 0.2, 0.5, 1, 2, 5, 10, 20, 40 and 80 μg/ml on HK-2 cells, respectively. Among them, the survival rate of HK-2 cells exposed to 40 μg/ml PAB (IC_50_ = 42.94 μg/ml) was (50.16 ± 1.82)%, whereas, showing no significant difference in comparison with that [(45.73 ± 3.52)%] upon the same concentration of ABZ-SO (IC_50_ = 21.22 μg/ml) (*p* = 0.2907) (Figure 7Ba). Similar to the response of HL-7702 cells, the number of HK-2 cells also decreased by crystal violet staining after treatment with 10 and 40 μg/ml PAB and ABZ-SO, respectively (Figure 7Bb).

### Hepatotoxicity and nephrotoxicity of PAB in mice

To assess the sub-acute hepatotoxicity and nephrotoxicity of PAB *in vivo*, Kunming mice were orally administrated with PAB for 6 weeks. Compared with the control group, the PAB group showed significant difference in serum direct bilirubin (DBIL) content with 79.68% decrease (*p* = 0.0015), total protein (TP) content with 1.1-fold increase (*p* = 0.0012), aspartate aminotransferase (AST) content with 1.1-fold increase (*p* = 0.0038), but the ABZ group showed significant difference in serum DBIL content with 79.69% decrease (*p* = 0.0015), indirect bilirubin (IBIL) content with 1.2-fold increase (*p* = 0.0063), TP content with 1.1-fold increase (*p* = 0.0027), alanine aminotransferase (ALT) content with 1.1-fold increase (*p* < 0.0001), AST content with 1.1-fold increase (*p* = 0.0373), alkaline phosphatase (ALP) with 1.2-fold increase (*p* = 0.0007). When compared with the PAB group, the ABZ group showed significance increase in serum IBIL content (*p* = 0.0408), ALT level (*p* = 0.0002), ALP content (*p* = 0.0005). Additionally, compared with the control group, the PAB group showed significant difference in serum creatinine (CRE) content with 1.2-fold increase (*p* = 0.0013) and blood urea nitrogen (BUN) content with 1.1-fold increase (*p* = 0.0329), and the ABZ group showed significant difference in serum CRE content with 1.2-fold increase (*p* = 0.0032) and BUN content with 1.1-fold increase (*p* < 0.0001). When compared with the PAB group, the ABZ group showed significance increase in serum BUN content (*p* = 0.0005; Table 3). In addition, no death and adverse reactions were observed in mice during treatment with PAB and ABZ.

**Table 3:** Changes of biochemical indexes in the serum of *E. multilocularis-*infected mice after treatment with PAB for 6 weeks (*n* = 5).

Test indexes	Control group (mean ± SD)	PAB group (mean ± SD)	ABZ group (mean ± SD)
TBIL (μM/L)	1.58 ± 0.10	1.71 ± 0.09	1.51 ± 0.22
DBIL (μM/L)	0.64 ± 0.05	0.51 ± 0.05^*^	0.51 ± 0.03^*^
IBIL (μM/L)	0.59 ± 0.05	0.62 ± 0.06	0.70 ± 0.01^*,#^
TP (g/L)	68.99 ± 2.18	77.18 ± 2.50^*^	76.35 ± 3.31^*^
ALT (U/L)	41.10 ± 1.08	42.53 ± 1.01	45.75 ± 0.15^*, #^
AST (U/L)	157.85 ± 8.22	176.70 ± 8.34^*^	170.82 ± 4.47^*^
ALP (U/L)	222.26 ± 9.06	219.78 ± 5.38	272.44 ± 24.89^*, #^
γ-GGT (U/L)	26.88 ± 2.06	26.59 ± 0.82	27.35 ± 0.60
CRE (μM/L)	12.93 ± 0.59	15.27 ± 1.21^*^	15.01 ± 0.15^*^
BUN (mM/L)	9.99 ± 0.18	10.52 ± 0.27^*^	11.48 ± 0.38^*, #^

PAB, pseudolaric acid B; TBIL, total bilirubin; DBIL, direct bilirubin; IBIL, indirect bilirubin; TP, total protein; ALT, alanine aminotransferase; AST, aspartate aminotransferase; ALP, alkaline phosphatase; γ-GGT, γ-gamma-glutamyl transpeptidase; CRE, creatinine; BUN, blood urea nitrogen. The data were assessed using one-way ANOVA with multiple comparisons.

^*^p < 0.05 (compared to the control group), and ^#^*p* < 0.05 (compared to the PAB group).

**Figure 8:**
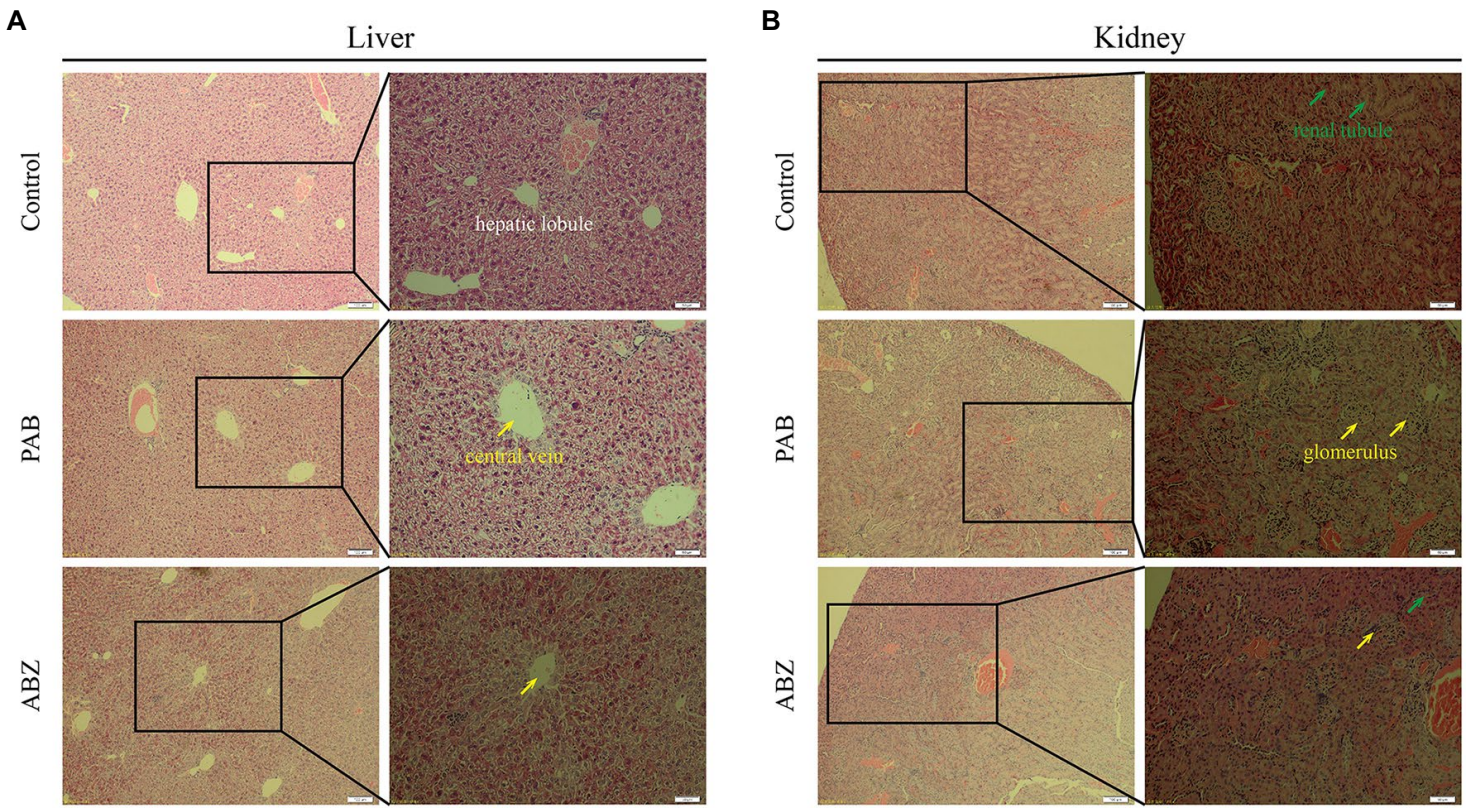
Hepatotoxicity and nephrotoxicity of PAB in mice. **(A)** Liver and **(B)** kidney histopathological images from Kunming mice treated with 40 mg/kg PAB and 100 mg/kg ABZ for 6 weeks (100 × and 200 × magnification), as detected by HE staining assay.

It was demonstrated that pathological changes of the liver and kidney tissues in the mice after treatment with PAB were examined by HE staining. In comparison with that in the liver of untreated mice, the liver in the mice treated with PAB or ABZ for 6 weeks did not show obvious pathological damage, such as infiltration of inflammatory cells in portal area, disorganization of hepatic lobule structure and ballooning or fatty degeneration of hepatocytes (Figure 8A). As well, compared with the liver of untreated mice, the kidney in PAB- or ABZ-treated mice did not show obvious pathological damage, such as renal tubular lesions, interstitial edema and accumulation of inflammatory cells (Figure 8B).

## Discussion

*E. multilocularis* metacestodes often reside in the liver of humans and plateau pikas, and is also termed “parasitic cancer” due to its tumor-like invasive growth pattern, causing a great concern in public health (Li et al., 2018; Gao et al., 2021). Currently, the drug of choice for AE is ABZ, which can limit rapid extension of *E. multilocularis* metacestodes, but it is difficult to meet curative goal for AE, and its long-term use produces strong adverse reactions (Liu et al., 2020b). Hence, development of new and effective anti-echinococcal drugs is imperative.

In the present study, the survival rate of *E. multilocularis* PSC exhibited a time- and dose-dependent decrease *in vitro* after treatment with PAB. Observed by SEM and TEM, we found that PSC upon exposure to PAB exhibited ultrastructural destructions, such as disappearance of syncytial layer, PAS stained materials and microvillus, collapse of parenchymae cells, and presentation of cytoplasm vacuolization, condensed chromatin and apoptotic bodies, which is similar to the pro-apoptotic activity of PAB in HCC cells (Gao et al., 2022). It is documented that *E. multilocularis* PSC has a duality: developing into an adult worm with sexual reproduction pattern in the definitive host, and initiating new cycles of asexual multiplication in the intermediate host, including development into microcyst, mature microcyst (production of cellular protrusions and brood capsules) and multiple cysts (production of abundant new PSC and multiple cysts) (Wang et al., 2016; Li et al., 2018). In the *in vitro* experiment, we observed that PSC grew into microcyst and completed a new cycle of asexual multiplication under the support of HepG2 cells, but this asexual cycle of PSC was blocked by PAB, showing collapse of microcyst, shrinking of germinal layer from the laminated layer after treatment with PAB. In addition, we found that after treatment with PAB, ALP activity in the culture supernatants of PSC *in vitro* was elevated significantly, which indicated a serious damage to *E. mutilocularis* metacestodes by PAB (Xin et al., 2020; Gao et al., 2021). At the same time, in the *in vivo* experiment, we also observed that the growth of *E. multilocularis* metacestodes was inhibited by PAB with a dose-dependent manner, evidenced by significant lower cyst weight after treatment with PAB. In short, PAB exhibits a strong parasiticidal effect on *E. multilocularis* larvae both *in vitro* and *in vivo*.

Previous study demonstrated that PAB exerts antitumor effects by activated apoptosis, autophagy and cell cycle arrest in certain types of cancer cells (Yu et al., 2008, 2016, Liu et al., 2020a). It was recognized that TGF-β1 signaling could trigger a variety of cellular responses, including inhibition of cell growth, migration, differentiation and apoptosis (Sanchez-Capelo, 2005). Later research exhibited that overexpressed TGF-β1 causes epithelial-mesenchymal transition (EMT), extracellular matrix (ECM) deposition and cancer-associated fibroblast (CAF) formation, which lead to fibrotic disease, and cancer (Peng et al., 2022). Thus, use of TGF-β1 chemical inhibitors as appears to be a new line of defenses against fibrotic disorders or cancer (Caja et al., 2018). Genome study indicated that *E. multilocularis* possesses TGF-β1, MAPK and Akt signaling pathways (Tsai et al., 2013; Zheng et al., 2013), beyond that, strong evidence suggest that during echinococccosis, the impaired host immune response is paralleled by an increased expression of TGF-β1 signaling components in periparasitic host cells and tissues (Nono et al., 2020). A later study on AE transplant treatment showed that through modulating the activity level of the TGF-β/Smad7 signaling pathway, liver fibrosis induced by *E. multilocularis* infection can be alleviated (Yang et al., 2022). In the present study, we observed apoptotic bodies in *E. multilocularis* PSC after treatment with PAB *in vitro*, referring apoptosis might be triggered. It was also demonstrated in our *in vitro* test that the TGF-β1 expression in the PSC was significantly reduced after treatment with PAB. On the other hand, we noted that the infection of *E. multilocularis* could induce over-expression of TGF-β1 protein and/or mRNA in the serum and liver of the mice, which was similar to the previous descriptions (Dissous et al., 2006; Brehm and Koziol, 2017). However, the over-expression of TGF-β1 in infected mice was significantly decreased after treatment with PAB, and meanwhile, the growth and proliferation of cysts was significantly inhibited *in vivo*. We thus deemed that the anti-parasite effect of PAB on *E. multilocularis* metacestodes was associated with the down-regulation of TGF-β1 signaling. Nevertheless, future study needs to elucidate the mechanism on how PAB regulate TGF-β1 signaling pathway to exert suppressive effect on the growth and proliferation of *E. multilocularis.* Current studies suggest that PAB is a promising immunosuppressive and anti-inflammatory agent candidate. It was evidenced that the derivative of PAB can promotes the production of Tregs and this inductive effect on Tregs production might be TGF-β1 dependent (Li et al., 2014). Some experiments demonstrated that TGF-β1 is secreted into the granuloma area around *E. multilocularis*, and the signaling may contribute to parasitic evasion of the host’s immunity (Zhang et al., 2008; Anthony et al., 2010). It was indicated that Treg/Th17 imbalance was observed at the middle and even late stage of *E. multilocularis* infection and it may be regulated by the TGF-β/Smad signaling pathway. TGF-β1 alone supports Treg cell expansion, TGF-β1 together with IL-6 promotes Th17 expansion (Gottstein et al., 2017; Wang et al., 2018). We expect future study on the action mechanism of PAB may include specific target effectors in TGF-β1 signaling possibly regulated by PAB, and other cytokine signaling pathway contributing to PAB’s suppressive effect on the growth and development of *Echinococcus* parasite.

PAB possesses potent cytotoxic effects to target cells; however, its cytotoxicity to the host is a research concern for future clinical application. To this end, a PAB derivative, hexahydropseudolaric acid B (HPAB) was synthesized and was demonstrated to be able to substantially reduce the cytotoxicity, while its inhibitory efficacy on T cell proliferation remains high (Li et al., 2014). Another experimental study indicated that PAB displays potent anti-chronic myeloid leukaemia (CML) cells activity and inhibits the growth of the tumors, but without being toxic to mice, suggesting PAB could be used as a potential treatment for CML patients (Liu et al., 2017; Choi et al., 2019; Jiang et al., 2020). In the present study, *in vitro* PAB showed inhibitory effect in human normal liver and kidney cells with IC_50_ = 25.29 and 42.94 μg/ml, which was in agree with the previous findings of the cytostatic effect of PAB on mouse liver and renal cells (Mei, 2011). Interestingly, the cytotoxicity of PAB to the two cells exhibited a weak effect superior to ABZ with IC_50_ = 3.71 and 21.22 μg/ml. Furthermore, we assessed the sub-acute hepatotoxicity and nephrotoxicity of PAB in the healthy mice, and found significant increase of serum IBIL, ALT, ALP and BUN level in mice treated with ABZ compared to PAB, which further indicates that PAB exerts a weaker hepatotoxicity and nephrotoxicity than ABZ. However, further studies are needed to elucidate whether chronic and genetic toxicity of PAB is also weaker than ABZ.

In conclusion, PAB exhibited evident suppressive effect on *E. multilocularis* metacestodes in our *in vitro* and *in vivo* tests. Meanwhile, we found that PAB reduces the expression of TGF-β1 protein and mRNA in *E. multilocularis* metacestodes *in vitro* and in infected mice, indicative of the anti-echinococcal effect may involve the reduction of the expression of TGF-β1. In addition, PAB exhibits lower cytotoxicity than ABZ-SO in the normal liver and kidney cell lines *in vitro*, and presents no sub-acute hepatic and renal toxicity in the healthy mice. Our study suggests the potential of PAB to be developed as a therapeutic agent. However, the chronic toxicity of PAB needs clarified. Moreover, future study is expected to further elucidate the mechanism of PAB exerting suppressive effect on the growth and proliferation of *E. multilocularis* metacestodes to gain insight into the passway of TGF-β1 signaling involved.

## Data availability statement

The original contributions presented in the study are included in the article/supplementary material, further inquiries can be directed to the corresponding authors.

## Ethics statement

The animal study was reviewed and approved by The Experimental Animal Ethics Committee of School of Basic Medical Sciences, Lanzhou University, and the Ethics Committee of the National Institute of Parasitic Diseases, Chinese Center for Disease Control and Prevention (Chinese Center for Tropical Diseases Research).

## Author contributions

TZ, HG, TJ, and WH designed the study. HG, XMo, BJ, YL, and BX carried out the experiments. HG, LH, and XMo analyzed data. TZ, WH, XMa, and JL provided experimental material. HG, TZ, and LH wrote the manuscript. HG, TZ, and ZF revised the manuscript. All authors contributed to the article and approved the submitted version.

## Funding

This work was supported by the National Key Research and Development Program of China (grant no. 2021YFC2300800 and 2021YFC2300802), NHC Key Laboratory of Parasite and Vector Biology (National Institute of Parasitic Diseases, Chinese Center for Diseases Control and Prevention; grant no. NHCKFKT2022-02), the Non-profit Central Research Institute Fund of Chinese Academy of Medical Sciences (grant no. 2019PT320004), NHC Key Laboratory of Echinococcosis Prevention and Control (grant no. 2021WZK1003) and the foundation of Shanghai Municipal Health Commission (grant no. 201940302). The funders had no role in the study design, data collection, data analysis, data interpretation, or the writing of this report.

## Conflict of interest

The authors declare that the research was conducted in the absence of any commercial or financial relationships that could be construed as a potential conflict of interest.

## Publisher’s note

All claims expressed in this article are solely those of the authors and do not necessarily represent those of their affiliated organizations, or those of the publisher, the editors and the reviewers. Any product that may be evaluated in this article, or claim that may be made by its manufacturer, is not guaranteed or endorsed by the publisher.
